# Supplementary material for: Resolving the full spectrum of human genome variation using Linked-Reads
Source: Genome Res. 2019 Apr;29(4):635–45. doi: 10.1101/gr.234443.118 (PMC6442396; doi:10.1101/gr.234443.118)
Supplement: Supplemental Material [file supp_gr.234443.118_Supplemental_longranger-2.2.zip › longranger-2.2.tar.bz2!/lr2.2-public/lib/stan/cmdstan/stan/doc/api/html/argument__probe_8hpp_source.html]

Stan: src/stan/gm/arguments/argument\_probe.hpp Source File


|  |  |
| --- | --- |
|  | Stan  2.5.0  probability, sampling & optimization |


- Main Page
- Related Pages
- Namespaces
- Classes
- Files

- File List
- File Members

All Classes Namespaces Files Functions Variables Typedefs Enumerations Enumerator Friends Macros Pages

- src
- stan
- gm
- arguments

argument\_probe.hpp

Go to the documentation of this file.

1 #ifndef STAN\_\_GM\_\_ARGUMENTS\_\_ARGUMENT\_\_PROBE\_\_HPP

2 #define STAN\_\_GM\_\_ARGUMENTS\_\_ARGUMENT\_\_PROBE\_\_HPP

3

4 #include <string>

5 #include <vector>

6 #include <sstream>

7

8 #include <stan/gm/arguments/argument.hpp>

9

10 namespace stan {

11

12  namespace gm {

13

14  class argument\_probe {

15

16  public:

17

18  argument\_probe(std::vector<argument\*>& valid\_args)

19  : \_arguments(valid\_args) {}

20

21  void probe\_args(std::stringstream& s) {

22

23  for (std::vector<argument\*>::iterator arg\_it = \_arguments.begin();

24  arg\_it != \_arguments.end(); ++arg\_it)

25  (\*arg\_it)->probe\_args(\*arg\_it, s);

26

27  }

28

29  protected:

30

31  std::vector<argument\*>& \_arguments;

32

33  };

34

35  } // gm

36

37 } // stan

38

39 #endif

40

argument.hpp

stan::gm::argument\_probe::argument\_probe

argument\_probe(std::vector< argument \* > &valid\_args)

**Definition:** argument\_probe.hpp:18

stan::gm::argument\_probe::probe\_args

void probe\_args(std::stringstream &s)

**Definition:** argument\_probe.hpp:21

stan::gm::argument\_probe::\_arguments

std::vector< argument \* > & \_arguments

**Definition:** argument\_probe.hpp:31

stan::gm::argument\_probe

**Definition:** argument\_probe.hpp:14


---

[ Stan Home Page ]

*© 2011–2014,
Stan Development Team.*
